# Prematurely ruptured dominant follicles often retain competent oocytes in infertile women

**DOI:** 10.1038/s41598-019-51551-9

**Published:** 2019-10-21

**Authors:** S. Teramoto, H. Osada, M. Shozu

**Affiliations:** 1 Natural ART Clinic at Nihonbashi, Tokyo, 103-6008 Japan; 2 Department of Reproductive Medicine, Postgraduate School of Medicine, Chiba University, Chiba, 265-6870 Japan

**Keywords:** Gonads, Physiology

## Abstract

Ovulation consists of a follicle’s rupture and subsequent oocyte extrusion, although there is a paucity of evidence regarding whether every follicle’s rupture is associated with extrusion of its oocyte. We examined this issue in a large-scale window-of-opportunity study by attempting aspiration of single dominant follicles that were found to have ruptured before a scheduled oocyte retrieval during *in vitro* fertilisation and embryo transfer treatment of infertile women. We were able to aspirate 587 of 1,071 ultrasonographically confirmed post-rupture dominant follicles from 1,071 women (i.e. one dominant follicle per woman) and retrieved 225 oocytes (oocyte recovery ratio: 43.4% of aspirated follicles), which yielded 28 live births (live birth ratio: 11.0% of retrieved oocytes). Interestingly, the live birth ratio for post-rupture dominant follicles was not statistically different from that achieved using regular pre-rupture aspiration of dominant follicles (1,085/8,977, 12.1%). These findings suggest that oocyte extrusion frequently does not occur after follicle rupture in infertile women undergoing *in vitro* fertilisation treatment, although the oocyte retained in the follicle can remain competent for use during that treatment.

## Introduction

In women, Graafian (dominant) follicles rupture and extrude mature oocytes during ovulation. It has been taken for granted that the follicle’s rupture is associated with oocyte extrusion, although there is a paucity of evidence that every follicle actually extrudes its oocyte. Two small-scale studies have described ruptured follicles that did not extrude their oocytes^1,2^, although those studies were based on experience with laparoscopic oocyte retrieval in the early 1980s during the advent of *in vitro* fertilisation (IVF) treatment. The first report described two sporadic cases in which laparoscopically confirmed ruptured follicles were needle aspirated and oocytes were successfully recovered^1^. The authors subsequently reported that an additional 13 oocytes were recovered using needle aspiration of 22 laparoscopically confirmed ruptured follicles during ovarian stimulation cycles^2^. These observations suggest that follicle rupture does not necessarily lead to oocyte extrusion, although we are not aware of any subsequent reports regarding this possibility. Furthermore, the introduction of ultrasonography for real-time follicle monitoring led physicians to abandon the needle aspiration of follicles if follicle rupture was detected, possibly due to the idée fixe that the ruptured follicle had completed oocyte extrusion.

Therefore, we hypothesised that ultrasonographically confirmed follicle rupture might not always be associated with oocyte extrusion, and conducted a large-scale window-of-opportunity observation study of prematurely ruptured follicles that were identified during normal IVF treatment with embryo transfer (IVF-ET) for infertile women. We also examined whether or not oocytes that remained entrapped within ruptured follicles also remained competent for fertilisation and whether they could produce live births as part of the IVF-ET treatment.

## Results

### Oocytes can be retrieved from post-rupture dominant follicles

Among the 1,071 post-rupture dominant follicles from 1,071 women (one dominant follicle per woman) that were identified ultrasonographically, the physician judged it possible to aspirate 587 follicles (54.8%), which yielded 255 oocytes (Table 1). Thus, the positive oocyte recovery ratio was 43.4% (255/587) for post-rupture dominant follicle aspiration, which was approximately one-half of the ratio for normal aspiration of unruptured dominant follicles (83.0%: 8,977/10,822).

**Table 1:** Results of oocyte retrieval from post-rupture follicle punctures and pre-rupture follicle punctures.

	Post-rupture follicle aspiration (n = 587)		Pre-rupture follicle aspiration (n = 10,822)		P^c^	RR	[95%CI]
	n	%	n	%			
Total oocytes retrieved (per total punctures)	255	43.4%	8,977	83.0%	<0.0001	0.18	[0.15–0.21]
Viable (per total oocytes)	206	80.8%	7,225	80.5%	n.s.	1.02	[0.75–1.39]
Damaged (per total oocytes)	49	19.2%	1,752	19.5%	n.s.	1.00	[0.76–1.33]
**Maturation status of viable oocytes on retrieval**							
Metaphase II (per viable oocytes)	194	94.2%	6,610	91.5%	n.s.	1.47	[0.84–2.66]
Metaphase I (per viable oocytes)	5	2.4%	526	7.3%	<0.01	0.30	[0.13–0.78]
Germinal vesicle (per viable oocytes)	7	3.4%	89	1.2%	<0.05	2.69	[1.30–5.57]
**Competency of oocytes**							
Oocytes inseminated^a^ (per total oocytes)	199	78.0%	7,087	78.9%	n.s.	0.95	[0.71–1.27]
Oocytes fertilized (per total oocytes)	167	65.5%	6,081	67.7%	n.s.	0.91	[0.70–1.17]
Good-quality blastocysts (per total oocytes)	68	26.7%	2,814	31.3%	n.s.	0.80	[0.61–1.05]
Clinical pregnancies (per total oocyte)^b^	40	15.7%	1,477	16.5%	n.s.	0.95	[0.68–1.32]
Live births (per total oocytes)	28	11.0%	1,085	12.1%	n.s.	0.90	[0.61–1.73]

^a^Equal to the number of oocytes that developed to metaphase II by the time of insemination.

^b^Ten good-quality blastocysts from post-rupture follicles and 378 good-quality blastocysts derived from pre-ruptured follicles had not been subjected to embryo-transfer by the end of the study period.

^c^χ^2^-test.

### Oocytes from post-rupture dominant follicles are competent

Among the 255 oocytes retrieved from post-rupture dominant follicles, 206 oocytes were viable and 194 oocytes (94.2%) were mature (metaphase II). These oocytes yielded 68 good-quality blastocysts and 28 live births. The live-birth ratio for post-rupture dominant follicle aspiration was 11.0% (28/255) of the total oocytes, which was comparable to the live-birth ratio for regular pre-rupture follicle aspiration (12.1%: 1,085/8,977) (Table 1).

Oocytes from post-rupture dominant follicles were slightly more mature than oocytes from pre-rupture dominant follicles (Table 1). However, post-rupture dominant follicles provided oocytes with slightly lower developmental potentials based on the ratios of good-quality blastocysts, clinical pregnancies, and live births. Nevertheless, these differences were not statistically significant (Table 1).

### Maturation status of cumulus cells in cumulus-oocyte complexes

The maturation status of the cumulus cells in the cumulus-oocyte complexes (COCs) was found to be significantly different when we compared post-rupture and pre-rupture dominant follicles (χ^2^ p < 0.001; Table 2). The post-rupture dominant follicles were more likely to have COCs with immature, over-mature, and other types of cumulus cell maturity. Furthermore, the post-rupture dominant follicles were less likely to have mature cumulus cells.

**Table 2:** Maturity of cumulus cells.

Cumulus status	Post-rupture follicles^a^ (%)		Pre-rupture follicles^a^ (%)		P^b^	Odds ratio	[95%CI]
Immature	26	(14.6%)	16	(3.4%)	<0.001	4.82	[2.52–9.23]
Mature	142	(79.8%)	450	(96.4%)	<0.001	0.15	[0.08–0.27]
Over-mature	6	(3.4%)	1	(0.25%)	<0.01	16.3	[1.94–136.00]
Others	4	(2.2%)	0	(0.0%)	<0.001	Not applicable	
Total	178		467				

^a^The number of cumulus oocyte complexes.

^b^χ^2^-test or Fischer’s exact probability test.

### Factors associated with oocyte recovery from post-rupture dominant follicles

We analysed factors that were associated with positive oocyte recovery from post-rupture dominant follicles (Table 3). The luteinising hormone (LH) levels on the triggering day were significantly associated with oocyte recovery, although the odds ratio was very close to 1.00, which suggested that the LH levels did not explain much of the variance. The use of minimal stimulation was also associated with a non-significantly increased likelihood of oocyte retrieval.

**Table 3:** Comparison of clinical characteristics between post-rupture follicles according to oocyte recovery status.

	Post-rupture follicle aspiration		Univariate			P^d^	Multivariate	
	Oocyte recovered^a^ (n = 255)	Oocyte negative^a^ (n = 332)	P^c^	Odds ratio	[95%CI]		Odds ratio	[95%CI]
Age (years)	38.6 ± 3.8^b^	38.9 ± 3.7^b^	n. s.					
**Ovary in which the dominant follicle grew**								
Right	143	185	n. s.	1.01	[0.84–1.21]			
Left	112	147						
**Use of ovarian stimulation**								
No stimulation (natural cycle)	130	147						
Minimal stimulation	125	185	n. s.	1.16	[0.97–1.40]			
**Hormone levels at day 3**								
Oestradiol (pg/mL)	49 ± 40	48 ± 26	n. s.					
LH (IU/L)	7.3 ± 3.5	7.4 ± 3.6	n. s.					
Anti-Mullerian hormone (ng/mL)	2.3 ± 2.2	2.0 ± 23	n. s.					
**Day of LH triggering**								
Duration from day 1 (days)	12.2 ± 5.0	12.1 ± 3.5	n. s.					
LH (IU/L)	25.8 ± 25.5	30.2 ± 26.9	<0.05			<0.05	0.99	[0.98–0.99]
Oestradiol (pg/mL)	335 ± 121	331 ± 127	n. s.					

^a^Number of cycles (equal to the number of dominant follicles).

^b^Mean ± SD.

^c^χ^2^-test or Wilcoxon signed rank test.

^d^Logistic regression analysis.

## Discussion

This study demonstrated that oocytes were retrievable from >40% of ultrasonographically confirmed post-rupture dominant follicles that could be aspirated during natural or minimally stimulated IVF-ET. Furthermore, we found that those oocytes were as competent as oocytes retrieved from pre-rupture follicles. These findings reinforce the concept that follicle rupture (fluid release) and ovulation (extrusion of the COC) are separate processes in a sequence of events.

The present study also confirmed that oocyte retention can occur in a large number of patients, which has previously been reported in controlled ovarian hyperstimulation cycles^1,2^, and extended this possibility to the rupture of a single dominant follicle. The oocyte recovery rate from the present study (43.4%) is lower than the previously reported recovery rate (13/22, 60%), which could be attributed to the different aspiration modalities (ultrasound-guided transvaginal aspiration vs. laparoscopically visualised direct aspiration). The most notable implication of the present study is that oocyte retention (extrusion loss) may occur even in spontaneous ovulatory cycles (natural cycles) that generate a single dominant follicle.

There are several possible explanations for the extrusion loss that we observed in the post-rupture dominant follicles. The first explanation involves a true failure of the ovulatory process, whereby oocytes are never extruded from the follicle. In this context, cumulus cell expansion is considered essential for COC release from the mural granulosa cell layer, although the precise mechanism of this release remains unknown^3,4^. After the LH surge, cumulus cells start to produce and secrete an extracellular matrix that consists of hyaluronan, proteoglycans, and proteoglycan-binding proteins, which facilitates cell detachment from the COC and release of the COC from the mural granulosa cell layer^5^. The association between cumulus cell expansion and COC release is also supported by findings from mouse models, as genetic disruptions of the extracellular matrix reduce ovulation frequency and increase entrapment of oocytes in the luteinised follicles, which suggests that cumulus cell expansion and COC release are inseparable co-dependent processes^3,6–10^. Our results revealed that a non-negligible number of post-rupture dominant follicles had less expanded cumulus cells (i.e. immature COCs) despite the accelerated maturation of the oocyte (i.e. a higher proportion of metaphase II). Therefore, we assume that limited expansion of cumulus cells, combined with accelerated oocyte maturation, may contribute to extrusion loss in prematurely ruptured dominant follicles. However, this cannot be a major mechanism, as most post-rupture COCs were composed of mature oocytes and mature cumulus cells. Intra-follicular ovarian pregnancy, in which a conceptus is located deep within the corpus luteum, might be a consequence of true failure of oocyte extrusion. In this scenario, the spermatozoon may enter the follicle through the stigma and fertilise the oocyte that is entrapped in the follicle, leading to *in situ* implantation^11–13^.

A second explanation for extrusion loss is a delay in the ovulatory process. In this context, the oocyte extrusion could proceed more slowly than we have assumed, or the extrusion process may resume long after the follicle’s rupture. However, a transvaginal ultrasonographic observation study of human chorionic gonadotropin-triggered follicles demonstrated that follicular fluid loss is a rapid process, with average times of 0.9 min for >70% follicle fluid loss and 6.1 min for complete fluid loss^14^. Furthermore, laparoscopic observations favour a similarly rapid process, as oocytes appear at the surface of the ovary or fallopian tube within 2–3 min after follicular rupture^15^. Other laparoscopic observations have indicated that follicular fluid release was more comparable to an explosive event than a slow oozing event^2^, and the authors speculated that the forceful outflow of follicular fluid contributed to the extrusion of the free-floating COC^2^. Thus, it is difficult to imagine that a diminished follicle, after follicular fluid release, resumes the process of extruding an oocyte that has been trapped in blood-containing fluids^2^. In addition, our observations revealed that the oocytes remained inside post-ruptured follicles despite the compressive pressure from a transvaginal ultrasonographic probe and needle puncture during the oocyte retrieval. This suggests that ruptured follicles are not vulnerable to external compressive pressure during oocyte extrusion. Therefore, based on previous reports and our experience, we do not believe that what we observed is simply a snapshot in an ongoing process of oocyte extrusion.

A third explanation for extrusion loss is the effects of exogenous manipulation, including the use of drugs for triggering the LH surge, which modulates some biochemical sequence(s) during the ovulatory process. Interestingly, extrusion loss occurred more often in cycles with lower LH levels at triggering than in cycles with higher LH levels at triggering (Table 3). This may suggest that inappropriate premature triggering may increase the chance of oocyte retention. Nevertheless, the odds ratio attributed to the LH level at triggering was as low as 0.99, which suggests that the timing of the triggering only had a minor effect on the likelihood of oocyte retention. In addition to its timing, the pharmacological actions of buserelin might increase the likelihood of extrusion loss, although we cannot comment on this possibility, as all women received buserelin in this study.

There are several limitations in this study. First, the study included IVF-ET cycles that were heterogeneous in terms of any ovarian stimulation. Another limitation is the possible selection bias that may have been introduced by only considering follicles that had prematurely ruptured before the scheduled oocyte retrieval. Moreover, our experience was limited to infertile women for whom IVF-ET was indicated. Therefore, our findings may not be replicated during cycles in which follicular rupture occurs later than 33–35 h after LH triggering, or in the cycles of fertile women. Moreover, the oocyte recovery ratio of post-rupture follicle (43.4%) might be overestimated, as quite a few cases were judged impossible to needle puncture, and the lowest possible estimate of the oocyte recovery ratio based on our data is 23.8% (255/1,071 of total post-ruptured follicles).

In conclusion, this study revealed that mature oocytes could routinely be retrieved from follicles that had prematurely ruptured in infertile women, which suggests that extrusion loss is not uncommon among these women. Future studies should determine whether extrusion loss only occurs during LH-surge manipulated cycles with premature follicle rupture in infertile women, or whether it occurs during the spontaneous ovulatory cycles of normally fertile women.

## Materials and Methods

### Ethical approval

The clinical application of oocyte retrieval from ultrasonographically identified ruptured follicles was initially approved by The Ethical Committee of Towako Clinics in 2010 (#2010-1). This study’s retrospective analysis of the related outcomes was also approved at a later date (#2014-3). Only patients who provided informed consent underwent the aspiration of post-rupture follicles with subsequent use of any retrieved oocytes in their IVF-ET treatment, in accordance with the Declaration of Helsinki. Furthermore, written informed consent was obtained from the patients for the use of the videos.

### Patients and study design

This opportunistic retrospective study included patients who underwent IVF-ET treatment in Shimbashi Yume Clinic between February 2011 and January 2016. The patients were eligible for natural-cycle or minimally stimulated IVF-ET, and completed 1–3 treatment cycles at the clinic. Most patients (76%) had previously experienced at least one IVF-ET treatment failure at other institutions. Thus, the patients desired an alternative method, and we offered them natural or minimally-stimulated cycles with re-assessment of their ovarian function. Couples with azoospermia were excluded, but couples with oligozoospermia were included. The patients’ clinical characteristics are shown in Table 4.

**Table 4:** Characteristics of the cycles with post-rupture or pre-rupture follicle aspiration.

	Post-rupture follicle aspiration^a^ (n = 587)	Pre-rupture follicle aspiration^a^ (n = 10,822)	Univariate analysis	
			P^c^	RR [95%CI]
Age (years)	38.7 ± 3.7^b^	38.4 ± 3.8	<0.05	
**Ovary in which the dominant follicle grows**				
Right	328	5,671	n. s.	1.14 [0.97–1.34]
Left	259	5,151		
**Use of ovarian stimulation**				
No stimulation (natural cycle)	310	4,656	<0.0001	1.45 [1.24–1.70]
Minimal stimulation	277	6,166		
**Hormone levels at day 3**				
Oestradiol (pg/mL)	48 ± 33	49 ± 29	n. s.	
LH (IU/L)	7.3 ± 3.6	7.8 ± 4.6	<0.05	
Anti-Mullerian hormone (ng/mL)	2.1 ± 2.32	2.3 ± 2.5	n. s.	
**Day of LH triggering**				
Duration from day 1 (days)	12.2 ± 4.2	12.4 ± 3.6	n.s.	
LH (IU/L)	28.3 ± 26.3	19.7 ± 16.7	<0.0001	
Oestradiol (pg/mL)	333 ± 124	332 ± 193	n. s.	

^a^Number of cycles (equal to the number of dominant follicles).

^b^Mean ± SD.

^c^χ^2^-test or Wilcoxon’s signed rank test.

**Figure 1:**
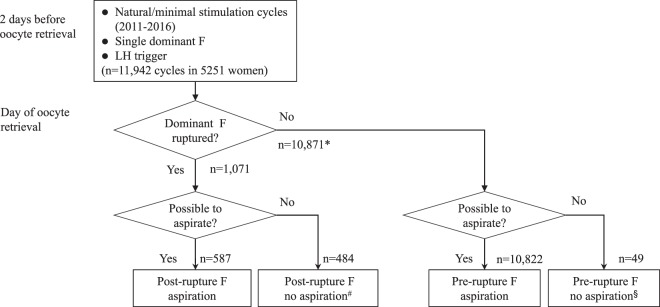
Flow chart of the study population. F: follicles; ^a^: number represents the number of cycles; ^b^: barely detectable follicles for puncture; ^c^: due to technical difficulty.

We identified 11,942 cycles (5,261 natural cycles and 6,681 minimally-stimulated cycles) in which a single dominant follicle had grown. Cycles in which ≥2 follicles grew to ≥11 mm on the day of triggering were not included in the analysis (Fig. 1). Among the 11,942 cycles, we identified 1,071 cycles with 1,071 dominant follicles (i.e. 1 follicle per cycle) that had unexpectedly ruptured, based on ultrasonographic confirmation, before the scheduled oocyte retrieval. Despite the follicle’s rupture, we performed post-rupture follicle aspiration and any resulting oocytes were used for IVF-ET. The IVF outcomes were compared to the regular IVF outcomes using oocytes that were normally aspirated from pre-ruptured follicles. Cycles in which the dominant follicle started to rupture at the oocyte retrieval (i.e. an ongoing decrease in follicular diameter was observed) were assigned to the pre-rupture follicle group.

### Follicle stimulation

For natural-cycle IVF, the patients only received buserelin acetate (300 µg) for LH triggering at 33–35 h before the scheduled oocyte retrieval. For minimally stimulated IVF, the patients received clomiphene citrate (25–50 mg per day) or letrozole (1.25 mg per day) for days 3–5 of the cycle, and also received buserelin acetate (300 µg) for the LH triggering.

### Oocyte retrieval

Follicle monitoring and triggering of the LH surge were performed as previously described^16^. The follicles were monitored using transvaginal ultrasonography (HI VISION Avius^®^; Hitachi Medical Corporation, Tokyo, Japan) and levels of oestradiol (E2) and LH were measured every day or every other day after day 10. Once a dominant follicle had reached a diameter of 16–18 mm and E2 levels of ≥190 pg/mL were detected, the buserelin acetate was administered nasally to trigger the LH surge and oocyte retrieval was scheduled for 33–35 h later. On the morning of the planned oocyte retrieval, follicles were examined using transvaginal ultrasonography to determine whether the dominant follicle remained intact (pre-rupture) or had prematurely ruptured (post-rupture). Ruptured follicles were identified based on the total or subtotal loss of echo-free follicular fluid contents, relative to the most recent examination.

If the ruptured follicle could be located within the ovary, based on the presence of an irregularly shaped central hypoechoic cavity, the physician (ST) punctured it using a 23-G needle while taking care not to puncture the small follicles around the ruptured dominant follicle. Representative needle aspirations are shown in Supplementary Videos 1 and 2. Most post-rupture dominant follicles had a 1–2-mm fluid-containing compartment that could still be punctured. After the puncture, the internal fluid was aspirated as thoroughly as possible by gently moving the needle tip around inside the space while repeatedly rotating the needle around its axis. Aspiration was performed using an automatic aspirator and negative pressure (−160 to −200 mmHg). Once back-flow had ceased, the needle was gently removed (without any additional puncture) and then flushed with a collection medium that contained heparin. The oocyte retrieval procedures were recorded and stored as ultrasonographic movies, and were later reviewed to exclude the possibility of inadvertent puncture of nearby small follicles^16^. The median time (± standard deviation) needed for aspiration of a post-rupture follicle was 33 ± 22 s. The aspirated fluid typically had a volume of approximately 0.1 mL and contained blood.

### IVF-ET

The recovered oocytes were released from the cumulus cells within 3 h after the retrieval. The maturity of the cumulus cells and oocytes was evaluated using a phase-contrast microscope. Metaphase II oocytes and metaphase I oocytes that developed to metaphase II stage within 10 h of the oocyte retrieval were inseminated using conventional IVF or intracytoplasmic sperm injection, based on the semen status and fertilisation outcomes from the previous IVF cycle(s). All embryos were cryopreserved when they had developed into good-quality blastocysts. The embryo was thawed and transferred during the following natural cycle or cycle with oestrogen and progestin supplementation^16^. All embryo transfers were single-embryo transfers, and were guided using transvaginal ultrasonography.

### Evaluation of COCs

Photographs of 178 COCs from post-rupture dominant follicles were available to evaluate the maturation status of the cumulus cells and intercellular substances. Five hundred photographs of COCs from the pre-rupture follicle were randomly selected as controls, and we found that 467 of those photographs were of sufficient quality to perform the evaluation.

The maturation status of the cumulus cells was classified into four levels (mature, immature, over-mature, and others) based on the criteria reported by Guelman and Patrizio^17^ (Supplementary Fig. S1). A COC with a sunburst-like corona radiata and a well-expanded cumulus was considered mature. A COC with compact cumulus cells in a cobble stone-like arrangement was considered immature. A COC with luteinised cells surrounded by massive gelatinous-like substances was considered over-mature. In over-mature cases, there was a reduced number of cumulus cells and it was not possible to distinguish between the corona and the cumulus. The “others” category includes COCs with few or no cumulus cells (denuded).

### Statistical analysis

The clinical pregnancy ratio and live-birth ratio were calculated using pregnancies that were positive for gestational sacs and pregnancies that yielded a live birth at ≥22 weeks of gestation, respectively. Wilcoxon’s signed rank test was used for the comparison of continuous variables. The χ^2^ test was used to evaluate the associations between categorical variables. If there were <5 expected categories for the χ^2^ test, the categories were dichotomised and the resulting 2 × 2 table was used for Fisher’s exact test. Logistic regression analysis was used to identify clinical factors that were associated with post-rupture follicle status. Differences with a p-value of <0.05 were considered statistically significant.

## Supplementary information

Video 1

Video 2

Supplementary information

Supplementary Figure S1

## Data Availability

All data generated or analysed during this study are included in this published article (and its Supplementary Information Files).
