# Supplementary material for: Prematurely ruptured dominant follicles often retain competent oocytes in infertile women
Source: Sci Rep. 2019 Oct 21;9:15041. doi: 10.1038/s41598-019-51551-9 (PMC6803670; doi:10.1038/s41598-019-51551-9)
Supplement: Supplementary file 3 — Supplementary information [file 41598_2019_51551_MOESM3_ESM.docx]

**Prematurely ruptured dominant follicles often retain competent oocytes in infertile women**

## S. Teramoto, H. Osada, M. Shozu

Supplementary information: Description of supplementary Videos 1 and 2.

Representative ultrasonographic videos taken during puncture of post-ruptured follicles, SI videos 1 and 2 of two women aged 38 and 35 years, respectively: Unruptured follicles around the post-ruptured follicle were of small follicle origin that measured ≤ 10 mm on the day of LH triggering, and remained unruptured following the LH surge. After each aspiration of the post-ruptured follicle, we reviewed all small follicles surrounding the post-ruptured dominant follicle and confirmed that they remained unruptured.
